# COVID-19 vaccination status in Germany: Factors and reasons for not being vaccinated (yet)

**DOI:** 10.3389/fpubh.2023.1070272

**Published:** 2023-02-13

**Authors:** Sebastian Sterl, Daniela Stelzmann, Nils Luettschwager, Lars Gerhold

**Affiliations:** ^1^Department of Mathematics and Computer Science, Institute of Computer Science, Interdisciplinary Security Research Group, Freie Universität Berlin, Berlin, Germany; ^2^Faculty of Life Sciences, Institute of Psychology, Psychology of Sociotechnical Systems, Technische Universität Braunschweig, Braunschweig, Germany

**Keywords:** COVID-19, vaccination status, vaccines, trust, Germany

## Abstract

**Introduction:**

The COVID-19 pandemic has demonstrated that effective vaccines constitute a central element of successful pandemic control. Although everyone in Germany has had the opportunity to receive a COVID-19 vaccine, some people remain hesitant or refuse to get vaccinated. To address this phenomenon as well as to examine the unvaccinated population more closely, the present study investigates (RQ1) factors explaining the COVID-19 vaccination status (RQ2) trust in different types of COVID-19 vaccines, and (RQ3) people's specific reasons for not getting vaccinated against COVID-19.

**Methods:**

We base our findings on a representative survey that we conducted in Germany in December 2021 with 1,310 respondents.

**Results:**

In response to the first research question, a logistic regression shows that trust in specific institutions (e.g., medical experts and authorities) is positively related to vaccination status, whereas trust in companies and COVID-19-related social and alternative media consumption decreases the likelihood of being vaccinated. Furthermore (RQ2), while vaccinated people trust mRNA-based vaccines (e.g., BioNTech), most unvaccinated people put greater trust in recently developed protein-based vaccines (e.g., Novavax), albeit on a low level. Finally, our study reveals (RQ3) that the most important reason why people choose not to get vaccinated is that they wish to make their own decisions about their bodies.

**Conclusion:**

Based on our results, we suggest that a successful vaccination campaign should address COVID-19 risk groups and lower income populations, increase trust in different public institutions and newly developed vaccines in advance, establish a multisectoral approach, and debunk fake news and misinformation. Furthermore, since unvaccinated respondents state that the desire to make their own choices about their body is the main reason why they have not gotten vaccinated against COVID-19, an effective vaccination campaign should emphasize the need for general practitioners who have a closer relationship with their patients who, in turn, trust their doctors.

## Introduction

By the end of February 2022, the COVID-19 pandemic had caused over 430 million infections with more than 6.3 million deaths worldwide (1). For Germany, approximately 27.3 million infections and more than 141,000 deaths had been reported by this time (2). With serious short- and long-term symptoms (e.g., long-term symptoms such as fatigue, headache, and attention disorder) (3), the virus continues to pose a serious threat to public health worldwide. Therefore, the implementation of preventive measures in society, such as social distancing, appears to be all the more important. However, vaccinations, described as “the most successful public health measure in history,” are the key preventive measure, saving approximately 2.5 million lives worldwide every year (4).

In terms of the prevention of COVID-19 infections, 20 November 2020, was seen as a turning point in the pandemic. On this date, the mRNA-based COVID-19 vaccine by BioNTech/Pfizer was first submitted for emergency use authorization in the United States (5). It was approved in Germany in late December 2020. By February 2022, further vaccines had been released in Germany, developed by Moderna (mRNA-based), Astra-Zeneca (vector-based), Johnson & Johnson (vector-based), and, more recently, Novavax and Valneva (protein-based). Although certain groups (e.g., high-risk groups) were prioritized initially, all such restrictions were lifted in June 2021 (6). When the COVID-19 vaccination program was first rolled out, demand was high, but it stagnated after a while (6). Despite a large-scale German vaccination media campaign in 2021 and 2022, a substantial portion of the population has refused the COVID-19 vaccination or remained hesitant toward it [as of 07 July 2021, ~9.2 million people aged 18 years or older (7)] — a phenomenon observed not only in Germany but also in many other countries [e.g., in the United States (8)].

The so-called vaccine hesitancy describes a refusal of or hesitancy toward vaccines despite their availability (9). The WHO includes it in its list of the top ten global health threats (10). In the context of the COVID-19 pandemic, many studies have examined factors explaining both vaccination hesitancy and willingness before a vaccine was even developed [e.g., (11, 12)]. The results indicate that vaccination willingness is linked to basic sociodemographic factors such as sex, age, and socioeconomic background [e.g., (11–13)], region [West Germany vs. East Germany: (6)] migration status (14), and belonging to a risk group and knowing people being hospitalized with COVID-19 (15). It is evident that trust in the state and its institutions is positively related to willingness to vaccinate [e.g., (16)], while right-wing views are related negatively (17). Moreover, various studies indicate that social media use represents a new factor concerning vaccination willingness (18). Social media channels are widely used by both governmental institutions to explain the effects of vaccination and anti-vax movements to spread misinformation, such as the claim that vaccination causes infertility (19).

Even though previous studies have generated broad knowledge of the factors of vaccination willingness, the abovementioned results were mostly compiled at a time when the vaccines were still being developed or their use was prioritized, as they were available only to risk groups or specific professions rather than to the public at large. Comparing the vaccination willingness in Germany during the prioritization phase with the actual vaccination rate after its lifting, data demonstrate a discrepancy of 22% between the willingness to get vaccinated and the actual vaccination rate (20, 21). In other words, the number of people willing to get vaccinated exceeded the actual vaccination rate. Therefore, we assume that measuring vaccination willingness cannot be equated with actual vaccination status. Against this background, the first research question (RQ1) examines the extent to which factors related to vaccination willingness also apply to vaccination status:

*RQ1: What individual factors explain the COVID-19 vaccination status?*

While many people recognize the benefit of COVID-19 vaccinations, there are individuals who distrust COVID-19 vaccinations. They do so for several reasons. Interestingly, there is higher trust on the whole in newly developed mRNA-based vaccines (e.g., BioNTech/Pfizer) than in other vaccine technologies (22), perhaps owing to the pioneering role of BioNTech/Pfizer, which received the first approval for a COVID-19 vaccine worldwide (23). Yet, the unvaccinated continue to mistrust this new vaccine technology (24). The most prevalent reasons include concerns about mRNA-based vaccination safety and the lack of long-term studies due to its relatively fast development and roll-out. Vaccinated people, however, are rather confident about the future of mRNA-based vaccines and medications and emphasize that this technology is safe because it has been explored for some time (24). Thus, compared with the unvaccinated population, vaccinated people put greater trust in these types of vaccines. In contrast, protein-based vaccines, such as the recently developed Novavax, are based on a “traditional” technology that has been used for influenza vaccines for a longer time. These protein-based vaccines could, therefore, be seen as a potential alternative for vaccination skeptics (25). A special campaign in Germany was implemented to educate the population about the different “new” and “old” vaccination technologies and to reduce mistrust [e.g., (26)]. This information was widely disseminated to the public, but it remains difficult to pinpoint to what extent knowledge about the technology or attitudes toward the manufacturers — based, for instance, on brand awareness — affects trust in the vaccines. To the best of the authors' knowledge, no study has investigated the trust of the vaccinated and unvaccinated populations in different specific types of COVID-19 vaccines. Findings could inform vaccination campaigns targeted toward these specific groups for the different vaccines. This includes the Valneva vaccine, which was not yet released during the survey period. Thus, we derive the second research question as follows:

*RQ2: To what extent does trust in COVID-19 vaccines differ between vaccinated and unvaccinated individuals?*

Although we can assume that trust is linked to vaccination status, previous studies have examined further reasons for vaccine hesitancy or refusal before vaccinations were available. These studies found that the most important reasons were largely of an internal nature, including concerns about safety, side effects, and the fast development of vaccines (27–29). External reasons, such as a lack of support from doctors, were less important for people not intending to get vaccinated (29). This raises the question of whether the reasons stated in previous studies remain constant over time or whether they evolve when vaccines become available. To the best of our knowledge, there is a lack of research analyzing the specific reasons and their relative importance for the unvaccinated population's choice in Germany not to get vaccinated. Therefore, it is important to investigate current reasons for vaccine hesitancy or refusal in greater detail to derive implications for addressing those not (yet) vaccinated more effectively:

*RQ3: What is the most important reason why people in Germany do not get vaccinated against COVID-19?*

## Materials and methods

### Procedure

To answer our research questions, we conducted a cross-sectional, online representative survey (in terms of age, sex, and German federal state) using an ISO-certified panel provider (Respondi, now called Bilendi, Germany). We conducted the survey across a stratified quota sample, interviewing an online panel of respondents in Germany from 20 December 2021 to 02 January 2022. In the first step, based on sociodemographic information, a random sample from the population of the online access panel is drawn. After this, a stratification or a quote module is used.

The interviews lasted for 26.8 min on average. Those who stated that they had not been vaccinated against COVID-19 were presented with a list of reasons for not being vaccinated. The whole study was conducted according to the Code of Ethics of the World Medical Association (30). Moreover, all subjects involved in the study gave their written informed consent. All information was collected anonymously.

### Participants

In total, 1,456 persons (18 years and older) completed the survey. However, the sample was restricted to 1,310 observations, since further analyses only included those with valid responses other than “I do not know” or “Answer refused” in all variables. As can be seen in Table 1, 49% were women and 51% were men; the age ranged from 18 to 74 (M = 45.71, SD = 15.04 years). Of the sample, 85% were located in West Germany and 15% in East Germany. The last column in Table 1 shows the true population values of sex, age group, and German state in 2021. Differences in age groups are due to the online representativeness of our sample.

**Table 1:** Descriptive statistics of all variables.

	**M**	**SD**	**Frequencies**	**Sample Percent**	**Percent German population 2021 (31–33)**
**Sex**					
Male			666	50.84	49.00
Female			644	49.16	51.00
**Age**	45.71	15.04			
**Age group**					
18 to 29 years			256	19.54	13.20
30 to 39 years			243	18.55	13.08
40 to 49 years			249	19.01	12.01
50 to 59 years			289	22.06	15.70
60 to 74 years			273	20.84	18.18
**Region**					
West Germany			1,111	84.81	84.97
East Germany			199	15.19	15.01
**Highest school degree**					
University/college			665	50.76	
No degree (yet)			5	0.38	
Low or high secondary			630	48,09	
Another			10	0.76	
**Household income (net)**					
€0-€1,499			290	22.14	
€1,500–€2,599			351	26.79	
€2,600–€4,999			542	41.37	
Over €5000			127	9.69	
**Migration status**					
No migration			1,082	82.60	
1st Generation			80	6.11	
2nd Generation			148	11.30	
**Children**					
No			643	49.08	
Yes			667	50.92	
**COVID-19 risk group**					
No			841	64.20	
Yes			469	35.80	
**Own COVID-19 infection**					
No			1,204	91.91	
Yes			106	8.09	
**Relative COVID-19 infected**					
No			651	49.69	
Yes			659	50.31	
**Trust in institutions**					
Politics	3.10	1.24			
Authorities/medical experts	3.59	1.23			
Hospitals/rescue workers	3.93	1.11			
Enterprise (e.g., food supply)	3.28	1.14			
State authorities	3.33	1.21			
Legal authorities	3.19	1.21			
**Political views**	5.01	1.63			
**COVID-19 media usage**					
Newspapers	1.79	1.72			
Tabloid media	0.75	1.34			
Public	2.79	1.68			
Private	1.94	1.74			
Official sources	1.90	1.51			
Science	1.35	1.48			
Social media	1.45	1.73			
Alternative media	0.56	1.23			
**Vaccinated against**					
**COVID-19**					
No			159	12.14	
Yes			1,151	87.86	

N = 1,310; M, mean; SD, standard deviation.

Approximately 51% of interviewees reported either a university or college entrance degree, while 48% achieved a middle-school or secondary-school diploma and 0.38% had no degree (yet). Most of the respondents reported their net household income to be between €2,600 and €4,999 (41%).

Of the sample, 83% had no so-called migration background, and 17% belonged either to the group of first-generation (6%) or second-generation immigrants (11%). A little over half of the respondents had children themselves (51%).

Notably, 36% stated that they belonged to a COVID-19 risk group, and 92% had not tested positive for COVID-19. However, 50% reported that a relative had tested positive for a COVID-19 infection. With 88%, our sample shows a slightly higher rate of vaccinated people aged 18 years or older than the official statistics in December 2021 with 84% (34).

### Measures

To determine the factors related to COVID-19 vaccination status (RQ1), we measured sociodemographic variables as well as those related to COVID-19 status, trust in institutions, political views, and COVID-19 media usage. On a descriptive level, we measured trust in different types of vaccines by vaccination status (RQ2) and by the most important reason for not being vaccinated (RQ3). Table 1 depicts the detailed descriptive statistics for all measures.

#### COVID-19 vaccination status

COVID-19 vaccination status was measured by the item “Have you already been vaccinated against COVID-19?” on a nominal scale including 0 (no) and 1 (yes). This variable serves as a dependent variable in the logistic regression model (RQ1) and as a variable differentiating the trust put in vaccines by those who are vaccinated and by those who are not (RQ2).

#### Sociodemographic variables

As sociodemographic characteristics, we measured common variables such as respondents' sex, age, and the German state in which they lived (grouped into the categories of “West Germany” and “East Germany”). Questions about the highest school degree received and the average net household income in Euro reflected the interviewees' socioeconomic status. For the questions about the highest school degree, the category “no degree (yet)” included both those who responded that they had no degree and those who had not yet received it. “Lower secondary school diploma” and “higher secondary school diploma” were grouped into the category “low or high secondary degree.” Finally, “advanced technical college entrance qualification, completion of a specialized secondary school” and “general or subject-related university entrance qualification” were grouped into the category “university/college entrance qualification,” while “other degree” remained the same (0.76%). Household income was split into the following four categories: “€0–€1,499,” “€1,500–€2,599,” “€2,600–€4,999,” and “Over €5,000.” Furthermore, we incorporated migration status in the categories “no migration background,” “first generation,” and “second generation,” which we generated on the basis of the respondents' and his/her parents' place of birth (“Were you born in Germany?”; “Were either of your parents born abroad?”; yes, no). Finally, we included information on whether the respondents had children [based on answers to the question “Do you have children? (yes, no)].

#### COVID-19 status variables

Furthermore, we assume that there is a link between belonging to a group at risk for a severe course of infection and vaccination status. Hence, we asked, “Would you say that you belong to a risk group?” (yes, no). Finally, we surveyed (a) the respondent's infection status [“Have you tested positive for COVID-19 (Coronavirus SARS-CoV-2)?”; yes, no] and (b) whether anybody in the respondent's family or acquaintances had tested positive for COVID-19 [“Have any individuals in your family or among your acquaintances tested positive for COVID-19 (Coronavirus SARS-CoV-2)?”; yes, no].

#### Trust in institutions and political views

We decided to survey trust in institutions and political views separately and in detail to investigate the relationship between specific public institutions and respondents' vaccination status and to derive implications and communication strategies for those factors that significantly impact people's tendency to be vaccinated. Political (right-wing) views are integrated as a single factor, since they may impact vaccination status independent of trust.

To assess trust in institutions, we used a battery of six items, measuring dimensions using a 5-point Likert scale ranging from 1 (do not agree at all) to 5 (fully agree). The topic was introduced as follows: “Now we will talk about your general attitudes toward dealing with COVID-19 (Coronavirus SARS-CoV-2). Please indicate the extent to which you agree with the following statements.” The six institutions include the following dimensions: “politics (federal government, state parliaments)”; “authorities and medical experts (e.g., Robert Koch Institute)”; “hospitals, rescue workers, and other aid organizations”; “companies, such as those in the food supply business”; “state authorities, such as the police and the public order office”; and “legal authorities, such as administrative or district courts.” All items were worded in the same way, beginning with the phrase “I trust that [respective institution] will do the right thing to protect me.” The scale was partially adapted from reference (35).

Political views were culled in response to the question, taken from reference (36), “Many people use the terms ‘left' and ‘right' to label different political attitudes. We present a scale from left to right. When you think of your own political views, where would you rank those views on this scale?” Responses were measured on a 10-point scale, ranging from 1 (left) to 10 (right).

#### COVID-19 media usage

To assess COVID-19 media usage, we employed a battery of eight items measuring different types of media with 6 points that ranged from 0 (never), 1 (less than once a week), 2 (once a week), 3 (several times a week), 4 (once a day), to 5 (several times a day), adapted from reference (37). To introduce the different types of media, we asked, “How often do you look for information about the COVID-19 pandemic on the following media?” The eight media types included “newspapers” (e.g., *Süddeutsche Zeitung*), “tabloid media” (e.g., *Bild*), “public media” (e.g., ARD, DLF), “private media” (e.g., RTL), “official sources” (e.g., Ministry of Health, Robert Koch Institute), “science” (e.g., journals, Nature), “social media” (e.g., Facebook), and “alternative media” (e.g., Ken FM, Nachdenkseiten).

#### Trust in COVID-19 vaccines

Trust in vaccines was measured by responses to the question, “How much trust do you have in the following COVID-19 vaccines?,” using a 5-point Likert scale, ranging from 1 (no trust at all) to 5 (very great trust) for six different types of vaccines, including the mRNA-based types BioNTech/Pfizer and Moderna, vector-based types Astra-Zeneca and Johnson & Johnson, and the recently developed protein-based types Novavax and Valneva.

#### Most important reasons for not being vaccinated

We measured the most important reason for not being vaccinated by presenting the interviewees with a list of 23 statements against COVID-19 vaccination (e.g., “Vaccines are not safe,” “I am fundamentally opposed to vaccinations,” or “My social contacts advised me against vaccination”). The participants were asked as follows: “You have indicated that you are currently not (yet) vaccinated against the Coronavirus. Below is a list of statements expressing why someone may not yet be vaccinated against Coronavirus. Please select up to five statements that most closely reflect your vaccination decision.” The participants ranked their choices in order of importance from 1 to a maximum of 5. In our analysis, we focused on the reasons that the participants selected as most important. These reasons were adapted from several scales [e.g., (27, 28, 38)].

### Statistical analyses

We used the statistical software Stata 17 SE to prepare and analyze the data. After data cleaning and checks, we conducted univariate analyses for all variables related to RQ1 and RQ2 (see Table 1). To analyze factors related to COVID-19 vaccination status in RQ1, we showed bivariate relationships between vaccination status and independent variables before conducting various multiple logistic regressions with the criterion of COVID-19 vaccination status. Therefore, all the factors described above were analyzed separately, leading to a final model comprising all variables related to the respondents' vaccination status. Since the dependent variable is dichotomous (0 “not vaccinated against COVID-19” and 1 “vaccinated against COVID-19”), we used a logistic regression model rather than a linear probability model (OLS regression model). The logistic function means that the range of predicted values lies between 0 and 1, although the linear combination of independent variables has no limits. Furthermore, the odds ratios are displayed, that is, the ratio of the probability of getting vaccinated and not getting vaccinated.

Therefore, we focused on the odds ratios, the standardized coefficients based on the logit coefficients, their direction and significance, and on the pseudo-R^2^ showing the goodness-of-fit. To analyze trust in vaccines as depicted in the responses to RQ2, we showed means of vaccine trust by vaccination status, using 95% coefficient plots and the unpaired *t*-tests to check statistical significance between unvaccinated and vaccinated persons. Finally, we addressed RQ3 by showing the univariate distribution of the most important reason for not being vaccinated.

## Results

### RQ1: Factors related to COVID-19 vaccination status

The second column in Table 2 displays the bivariate relationship between each independent variable and the criterion of vaccination status, using a simple logistic regression model and the unadjusted odds ratios of every single predictor based on our data. For the sociodemographic variables, both age and higher income relate positively to being vaccinated, while respondents living in East Germany (compared with West Germany) and those with no school degree (compared with college/university degree) are less likely to be vaccinated. By contrast, there is no relationship between the respondents' vaccination status and either their migration status or whether or not they have children. All COVID-19 status variables correlate significantly with the participants' vaccination status. While belonging to a COVID-19 risk group and knowing someone infected with COVID-19 are factors that relate positively to being vaccinated, a respondent's own COVID-19 infection relates negatively to being vaccinated. While all variables depicting trust in institutions increase the likelihood of a COVID-19 vaccination, the level of right-wing political attitude decreases this probability. The likelihood of getting the COVID-19 vaccination also depends on the type of media a respondent consumes. While those who consume newspapers, public and private media, as well as official sources are significantly more likely to be vaccinated, the likelihood is low for those consuming more social and alternative media.

**Table 2:** Logistic regression results using vaccination status (0 ”not vaccinated against COVID-19,” 1 ”vaccinated against COVID-19”) as the criterion.

		**M1: Sociodemographic**	**M2: COVID−19 status**	**M3: Trust in institutions**	**M4: COVID−19 media usage**	**M5: All**
**Factor**	**Unadjusted odds ratios (unadjusted logit coefficient)**	**Adjusted odds ratios (standardized coefficient based on adjusted logit coefficient)**				
Female (ref. = male)	0.949 (−0.053)	1.080 (0.020)				0.756 (−0.051)
Age	**1.021** ^***^ **(0.021)**	**1.028** ^***^ **(0.220)**				1.008 (0.046)
East Germany (ref. = West Germany)	**0.391** ^***^ **(−0.938)**	**0.420** ^***^ **(−0.163)**				**0.468** ^**^ **(−0.100)**
Highest school degree						
University/college	Ref.	Ref.				Ref.
No degree (yet)	**0.083** ^**^ **(−2.483)**	**0.062** ^**^ **(−0.090)**				**0.040** ^*^ **(−0.073)**
Low/high secondary	0.849 (−0.164)	0.751 (−0.075)				0.803 (−0.040)
Another	1.127 (0.119)	0.965 (−0.002)				0.241 (−0.045)
Household income (net)						
€0-€1,499	Ref.	Ref.				Ref.
€1,500-€2,599	1.132 (0.124)	1.060 (0.013)				0.852 (−0.026)
€2,600-€4,999	**1.563** ^*^ **(0.447)**	1.472 (0.100)				0.977 (−0.004)
Over €5,000	**2.732** ^*^ **(1.005)**	**2.362** ^*^ **(0.133)**				**3.459** ^*^ **(0.135)**
Migration status						
No migration	Ref.	Ref.				Ref.
1st generation	1.096 (0.092)	1.235 (0.027)				2.443 (0.078)
2nd generation	1.004 (0.004)	1.116 (0.018)				1.139 (0.015)
Children (ref. = no)	1.153 (0.142)	0.804 (−0.057)				1.194 (0.032)
COVID-19 status						
Risk group (ref. = no)	**2.536** ^***^ **(0.931)**		**2.546** ^***^ **(0.237)**			**2.032** ^**^ **(0.125)**
Own infection (ref. = no)	**0.561** ^**^ **(−0.578)**		**0.525** ^*^ **(−0.093)**			0.626 (−0.047)
Relative infected (ref. = no)	**1.542** ^*^ **(0.433)**		**1.694** ^**^ **(0.139)**			1.104 (0.018)
Trust in institutions						
Politics	**2.771** ^***^ **(1.019)**			1.273 (0.127)		**1.471** ^*^ **(0.175)**
Authorities/medical experts	**3.071** ^***^ **(1.122)**			**2.386** ^***^ **(0.458)**		**2.354** ^***^ **(0.387)**
Hospitals/rescue workers etc.	**2.333** ^***^ **(0.847)**			**1.488** ^***^ **(0.187)**		**1.377** ^*^ **(0.130)**
Companies (e.g., food supply)	**1.648** ^***^ **(0.500)**			**0.717** ^**^ **(−0.162)**		**0.743** ^*^ **(−0.124)**
State authorities	**2.518** ^***^ **(0.924)**			1.220 (0.103)		1.087 (0.037)
Legal authorities	**2.144** ^***^ **(0.763)**			0.784 (−0.125)		0.780 (−0.110)
Political views	**0.801** ^***^ **(−0.222)**			0.915 (−0.062)		0.887 (−0.072)
COVID-19 media usage						
Newspapers	**1.287** ^***^ **(0.252)**				1.160 (0.113)	1.062 (0.038)
Tabloid media	0.921 (−0.083)				0.928 (−0.044)	1.004 (0.002)
**Factor**	**Unadjusted odds ratios (unadjusted logit coefficient)**	**Adjusted odds ratios (standardized coefficient based on adjusted logit coefficient)**				
Public media	**1.616** ^***^ **(0.480)**				**1.442** ^***^ **(0.274)**	1.131 (0.076)
Private media	**1.175** ^**^ **(0.161)**				1.078 (0.058)	0.986 (−0.009)
Official sources	**1.490** ^***^ **(0.399)**				**1.483** ^***^ **(0.265)**	1.087 (0.046)
Science	1.052 (0.051)				0.890 (−0.077)	0.862 (−0.081)
Social media	**0.882** ^**^ **(−0.126)**				**0.859** ^*^ **(−0.117)**	**0.852** ^*^ **(−0.102)**
Alternative media	**0.618** ^***^ **(−0.482)**				**0.579** ^***^ **(−0.299)**	**0.680** ^***^ **(−0.173)**
Observations	1,310	1,310	1,310	1,310	1,310	1,310
Pseudo-R^2^		0.055	0.036	0.287	0.209	0.393

* p < 0.05,

** p < 0.01,

*** p < 0.001.

Based on our first research question, we performed five logistic regression models to investigate to what extent the individual dimensions of sociodemographic background, COVID-19 status variables, trust in institutions and political views, and COVID-19 media usage related to the respondents' COVID-19 vaccination status. Table 2 shows all models of the logistic regression analyses.

Model 1 comprises all sociodemographic variables and demonstrates that age and a household income >€5,000 increase the likelihood of being vaccinated, while residence in East Germany and a lack of school degrees decrease the likelihood. All other factors, such as sex, migration background, and having children, show no relationship to the respondents' vaccination status. However, the explanatory power of all the sociodemographic variables is rather low (pseudo-R^2^ = 0.06).

Model 2 comprises variables directly related to COVID-19. Belonging to a risk group as well as knowing people with a COVID-19 infection increase the likelihood of being vaccinated, whereas one's own infection decreases it. With a pseudo-R^2^ of 0.04, the COVID-19 status variables are correlated with vaccination status very weakly.

Trust in institutions as well as political views, as shown in model 3, have a rather high explanatory power (pseudo-R^2^ = 0.29). Trust in medical experts and authorities as well as in hospitals, rescue workers, and aid organizations increases the likelihood of being vaccinated, whereas trust in companies makes vaccination is less likely. Political views also do not relate to vaccination status compared with the unadjusted model.

According to model 4, those who turn to public and official media to inform themselves about COVID-19 are more likely to be vaccinated. By contrast, those using social media and alternative information channels are less likely to be vaccinated. With a pseudo-R^2^ of 0.21, COVID-19 media usage has the second highest explanatory power after trust in institutions.

Finally, model 5 comprises all variables related to vaccination status and shows a high model fit of pseudo-R^2^ = 0.39. In terms of sociodemographic background, sex, age, and migration status do not correlate significantly with vaccination status. However, respondents from East Germany and people without a school degree are less likely to be vaccinated, while those with a household income of €5,000 or more are more likely to be vaccinated. Furthermore, belonging to a risk group increases the likelihood of being vaccinated against COVID-19, while both the respondents' own and a relative's COVID-19 infection status show no effect. Concerning attitudes toward institutions, trust in political institutions, medical experts and authorities, hospitals, rescue workers, and aid organizations increases the likelihood of being vaccinated, while trust in companies is negatively related. However, trust in state and legal authorities and political views show no correlation with being vaccinated against COVID-19. Where COVID-19 media usage is concerned, consuming information from social media and alternative media corresponds negatively with being vaccinated against COVID-19, while consuming information from newspapers, tabloids, public and private media, and official sources or science does not relate to vaccination status.

### RQ2: Trust in COVID-19 vaccines by vaccination status

Overall, vaccinated people have a significantly higher trust in all types of vaccines than unvaccinated people [BioNTech: *t*_(1234)_ = −27.65, *p* < 0.001; Moderna: *t*_(1233)_ = −25.11, *p* < 0.001; Astra-Zeneca: *t*_(1220)_ = −12.97, *p* < 0.001; Johnson & Johnson: *t*_(1193)_ = −10.95, *p* < 0.001; Novavax: *t*_(689)_ = −8.02, *p* < 0.001; Valneva: *t*_(462)_ = −4.48, *p* < 0.001].

Vaccinated people have the highest trust in BioNTech, whereas unvaccinated people trust Novavax the most. By contrast, vaccinated people express the lowest trust in Valneva, while those who are not vaccinated trust Astra-Zeneca the least.

**Figure 1:**
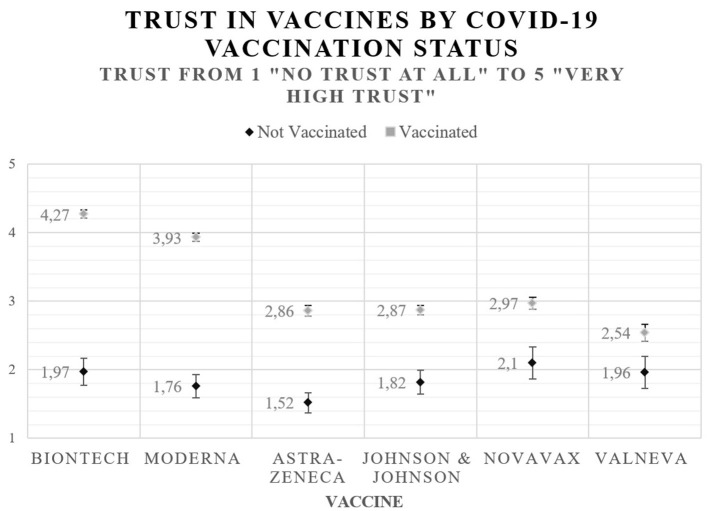
Trust in COVID-19 vaccines.

All in all, those who have been vaccinated trust mRNA-based vaccines the most, whereas those who have not been vaccinated put their highest trust in the recently developed protein-based vaccines, which use the same technology as influenza vaccines. Regarding unvaccinated people, our data reveal no significant differences between trust in traditional vaccines and other types of vaccines, except for Astra-Zeneca. Figure 1 depicts trust in different types of COVID-19 vaccines.

### RQ3: The most important reason for not being vaccinated against COVID-19

**Figure 2:**
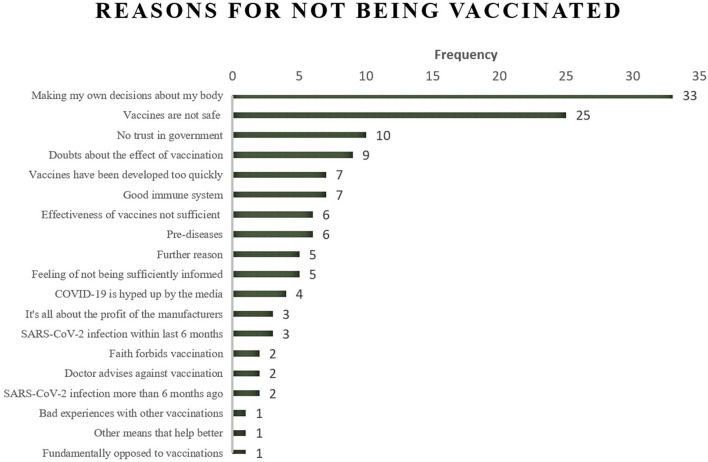
Most important reason for not being vaccinated against COVID-19 (*N* = 132). Not vaccinated respondents could state up to five reasons. Here the most important reason is displayed.

Among unvaccinated individuals, the wish to “make my own decisions about my body” plays a major role (*N* = 33), followed by safety concerns (“Vaccines are not safe,” *N* = 25) and “lack of trust in government” (*N* = 10). To explain the last three reasons, only three people in the total state that they had a “bad experience with other vaccinations” (*N* = 1), that “other means help better” (against COVID-19, *N* = 1), or that they are “fundamentally opposed to vaccinations” (*N* = 1). Figure 2 shows the most important reason for not being vaccinated.

## Discussion

### RQ1: Factors related to COVID-19 vaccination status

Our first research question addresses individual factors that may explain the COVID-19 vaccination status. In many regards, our results (based on model 5) identify previous findings on factors related to vaccination willingness. For instance, our results document a moderate correlation between socioeconomic factors, such as income, and vaccination status (11–13). Interestingly, these correlations are especially prevalent in margin categories (e.g., low income is related to low vaccination status). Current scholarship often explains these relationships by pointing to a lack of understanding of vaccine importance and insufficient access to medical care (39). It remains unclear to what extent the latter applies to a sample in Germany, where access to healthcare is comparatively secure. Nevertheless, it seems essential to educate the public about the advantages of vaccination in general and to emphasize that vaccinations are accessible to all and free of charge.

Contrary to previous research on vaccination willingness, our analyses do not show that sociodemographic background, including factors such as age, sex, and migration status, plays a significant role in predicting vaccination status (11–14, 39). Regarding age and vaccination status, it seems plausible that the risk of the severe course of disease eliminates this relationship. Hence, we assume that belonging to a risk group relates to both vaccination willingness and status more than age does. For sex and migration status, none of our models documented a link to vaccination status. As the SAGE vaccine hesitancy determinants matrix (9) suggests, individual and group factors, such as perceived risk and benefits, influence vaccination status, which may explain the non-significant correlation in the present study. Thus, future research should focus on these contradicting results by considering additional factors (e.g., risk perception and language barriers).

Apart from socioeconomic and sociodemographic factors, our study partly replicates results from previous surveys. Thus, it corresponds to previous findings [e.g., (15)] that people who belong to a COVID-19 risk group have a higher vaccination rate. This finding is not surprising, since those in a risk group have a high likelihood of a severe course of infection, hospitalization, and death. This raises the concern that people over 60 years of age are at higher risk of serious infection, due to older age and related comorbidity (40) alone. However, in this age group, there is a massive immunization gap of 1.9 million people in Germany [July 2022, (7)]. Although these people do not see themselves as being at risk, we cannot deny the objective risk of advanced age. Thus, it is important to address unvaccinated people in this risk group both by encouraging general practitioners to educate them individually and by launching public campaigns that inform older people about the immediate risks caused by an infection.

In contrast to belonging to a risk group, which seems to be a reliable factor for vaccination status and willingness, the results regarding respondents' own infection status and knowing someone who was or is infected are inconsistent [e.g., (11, 15)]. Future research should investigate this potential relationship in greater detail, for instance, by focusing on the role of the severity of the course taken by the disease, the impact of infection waves (Delta vs. Omicron variant), the number of subsequent infections, and the strength of social tie to the infected person.

Corresponding with the official COVID-19 statistics, people from East Germany are less likely to be vaccinated than those from West Germany (6). This finding depicts a new phenomenon and differs from the correlation between residence and other disease-related vaccinations (e.g., tetanus or diphtheria), which shows higher vaccination rates for East Germans than for West Germans (41). One explanation may be that in East Germany, there is widespread support of the right-wing populist party AfD (Alternative for Germany), which is skeptical of or refuses COVID-19 measures, such as wearing masks (42), a fact associated with a lower trust in public and state institutions. Thus, fostering trust in public institutions, debunking populist parties' false claims about the COVID-19 measures, and locally targeted health campaigns may help further increase the vaccination rate in the regions concerned.

Furthermore, trust in institutions shows the most significant correlation with vaccination status. A novel finding of our study is that the more people put trust in companies (e.g., food supply), the less likely they are to get vaccinated. One explanation might be that as part of the private sector in the free market, companies tend to symbolize a libertarian point of view, suggesting that the decision to get vaccinated is related to an individualistic, liberal attitude fostering vaccination. This means it is more a personal, private choice rather than a matter of public health. To resolve this association, private companies should be actively involved in a vaccination campaign to increase the percentage of the vaccinated population. Therefore, Dhama et al. (43) proposed a multisectoral approach, defined as an alliance between various agencies from the public and private sectors, to build long-lasting trust in vaccines. In Germany, more than 150 companies advertised a vaccination against COVID-19 together and, therefore, temporarily changed their branding [e.g., chocolate manufacturer Rittersport or supermarkets like LIDL; (44)]. Such strategies could be implemented more extensively to raise vaccination rates in the future.

In contrast to previous research [e.g., (17)], political views as measured on a left-right political scale have no significant relationship to vaccination status. This may be so because trust in public institutions eliminates that relationship. Thus, being politically right-wing decreases institutional trust, which, in turn, relates to being vaccinated negatively. Consequently, building trust in public institutions is more important to the objective of reaching a high vaccination rate, as the next section explains in further detail.

In line with previous research, trust in politics, in hospitals/rescue workers, and, above all, in medical experts and authorities (11, 16, 17, 45) increases the likelihood of getting vaccinated. It is evident that trust plays an important role in ensuring compliance with public health measures in general (45), is related to prevention measures, such as getting vaccinated, in particular, and thus constitutes a highly relevant resource that must be maintained by public institutions. For instance, to cope with a crisis and maintain trust, these institutions must have a stringent communication strategy, which was lacking during the pandemic in public perception (46) (p. 55). Hence, building trust in public health institutions is vital to the objective of increasing the overall vaccination rate. It could be achieved by clear, target-oriented, and effective communication addressing those not fully convinced by the vaccination yet.

As Yang and Huang (47) hold, health communication that combines high-quality information with a traditional communication style, e.g., through banners and posters, leads older people in particular to develop greater trust in science and health professionals. However, social media as a means of communication can decrease trust, demonstrating that the quick spread of misinformation on social media negatively influences people's opinions (48). Corroborating the findings of previous research [e.g., (45)], our study shows that those who get COVID-19 information from social and alternative media are less likely to be vaccinated. There is an especially high negative correlation to vaccination status for those who consume the alternative media on COVID-19 that spread on social media channels such as Telegram. This is due to the fact that fake news, scientific misinformation, and conspiracy theories about health risks are widely distributed through alternative media (49) and shared on social media such as Telegram (50), which then raises these media consumers' concerns about public health measures such as vaccinations. By contrast, our final model suggests that traditional media consumption has no impact on vaccination status. Given that social media consumption is likely to have a negative impact on vaccination status, it is advisable that content from traditional media be disseminated on social media. Although this is already partly the case, future studies should test the strategies for communicating traditional media on social media so as to generate the greatest possible benefit for reaching sufficiently high vaccination rates.

The implications of this study are 2-fold. First, countermeasures, such as online fact checks, marking false statements, and social and alternative media surveillance and regulations, should be increased to both debunk false claims about the COVID-19 vaccinations and alleged side effects and communicate clearly and openly with people who are hesitant to get a COVID-19 vaccine. For instance, Facebook uses tools to alert users that they have read a post containing incorrect information and to make these posts less visible (51). Second, since our study shows that trust in medical experts and authorities is most closely associated with a higher likelihood of getting vaccinated, relevant institutions should reclaim the high ground of COVID-19 coverage on social media to “turn around” the relationship between social media and vaccination status as shown in our results above.

### RQ2: Trust in COVID-19 vaccines by vaccination status

Since we can assume that trust in vaccines is related to vaccination status, our second research question examines the difference of trust in the COVID-19 vaccines between vaccinated and unvaccinated individuals. Our results demonstrate that those who are vaccinated show higher trust in all vaccines, especially in mRNA-based vaccines, than those who are not vaccinated. This is because most people in Germany received mRNA-based vaccines (6) and so place the greatest trust in these vaccines in order to, for instance, reduce cognitive dissonance (52). In line with the rather negative media coverage (53), Astra-Zeneca is rated badly compared with other vaccines. Such a correlation is not a new phenomenon and can also be observed when persons are exposed to health media in mass media, decreasing patients' belief in the benefits of medication (54). This phenomenon also emerged for other vaccines, such as that against human papillomavirus (HPV) (55). Furthermore, people tend to place greater weight on negative media reports (56), which could lead to a more elevated risk perception of possible side effects and so to decreased trust.

The vaccines Novavax and Valneva, based on a “traditional” technology used for influenza vaccines, are often seen as an alternative for unvaccinated people, who trust these vaccines the most, and thus as a way to increase the vaccination rate (25). However, trust in these vaccines is still low and does not differ from other vaccines so much, which is also reflected in the fact that Novavax has not increased the demand for vaccinations significantly since its roll-out in February 2022 (57). According to the German Minister of Health, this might be due to fake news on social media claiming Novavax would cause cancerous tumors, among other things (58). Such claims spread faster than public vaccination campaigns for the use of Novavax. This demonstrates the need to implement a vaccination campaign for the other protein-based vaccine Valneva before its release so as to build trust long in advance and debunk false claims as soon as they start to spread even on a small scale.

Although the results of our survey demonstrate a relationship between trust and vaccination status, it remains unclear to what extent this trust is generated by knowledge about the vaccine and attitudes toward the manufacturer arising from brand awareness. Recapitulating the massive media presence of the manufacturers and the intensive coverage of side effects [e.g., Astra Zeneca; cerebral venous thrombosis (53)], it seems plausible that trust cannot be traced back solely to knowledge about the vaccines. Therefore, future studies should investigate factors (e.g., knowledge and brand awareness) that may explain the relationship between trust and vaccination status.

### RQ3: The most important reason for not being vaccinated against COVID-19

Our last research question aims to identify the most important reason for not being vaccinated against COVID-19. Our analyses revealed that the first reason is the respondents' wish to make their own decisions about their bodies, followed by doubts about vaccine safety. This result partly contradicts research on people's reasons for refusing vaccination when vaccines were not available yet. These studies cited concerns about safety, side effects, and the vaccines' fast development as people's most prominent reasons against vaccination (27–29), demonstrating a move from “general” safety reasons to highly individual reasons concerning the conditions of one's own body. Moreover, a lack of trust in the government fully corresponds to previous research (16) and findings in this study on factors related to vaccination status as described above.

In other words, the issue at hand mainly concerns decisions about and control of one's own body, which is relatively difficult to address in public vaccination campaigns compared with the safety worries expressed in former studies. Our study reveals a more affective and very individual statement about the desire to keep full control of one's own body and the freedom to do so. Because public vaccination campaigns on traditional or social media platforms may fail to address that highly individual and ethical reason, a person who knows the other's specific body condition well enough is central: Therefore, general practitioners play a key role since doctors know their patients' body condition best, while patients “[…] are likely to establish trust with known health professionals, as their experience of that person increases” (59) (p. 2). Hence, healthcare providers' recommendations have a strong positive relationship to vaccination willingness [e.g., (60) for hepatitis B vaccination]. At the same time, studies have shown that healthcare practitioners' training is essential in treatment to counteract existing fears about vaccination by informing and involving patients in the process of decision-making. Doing so increases the adherence to medical recommendations [e.g., (61)]. Conversations with the general practitioner, called “second-tier” or informal support, are essential to making an informed decision about whether to get vaccinated (62) and are thus an effective element in increasing the vaccination rate against COVID-19. According to Bartoš et al. (63), while ~90% of medical doctors trust COVID-19 vaccines, most respondents from the general public believe that only half of the doctors trust the vaccines. Future vaccination campaigns should address this misconception by actively integrating medical doctors.

### Limitations

Due to research and survey design, our study is subject to several methodological limitations. First, our data were generated using an online panel. Even though we aimed to fully represent the German population, our data are only online representative of sex, age groups from 18 to 74 years, and federal state. Moreover, our data did not gather specific groups, such as people younger than 18 years or older than 74 years or those with no online access at all, which is clear from the demographic differences between our sample and the actual German population in 2021 as shown in Table 1. Therefore, future studies should also conduct telephone (CATI) or personal interviews (CAPI), in order to minimize these restrictions. Furthermore, our study was designed as a cross-sectional survey to collect data. Consequently, interpretations of the data are linked to the time and location of data collection and are thus subject to a certain reactivity, since surveys concerning vaccination status depend on dynamic contextual factors. While the cross-sectional design prevents us from drawing other causal conclusions, repeating this survey using longitudinal data with the same sample could allow for more causal statements.

## Conclusion

This study analyzes factors related to the COVID-19 vaccination status as well as trust in specific types of COVID-19 vaccines and the most important reason for not being vaccinated at a given point in time, when everybody has had the opportunity to receive a vaccine that protects them from a severe course of illness, long-term effects, or hospitalization caused by a viral infection. The study shows that it is vital, from a public health perspective, to address COVID-19 risk groups and lower income populations, elevate trust in different institutions and newly developed vaccines in advance, establish a multisectoral approach, and implement campaigns to debunk misinformation. However, unvaccinated people's lack of trust in all COVID-19 vaccines, along with the most prominent reason cited against vaccination of wanting to make one's own decisions for one's body, suggests that general practitioners should be involved in an effective vaccination campaign, while scientific predictions of rising infection numbers (64) and future virus variants should play a role in developing efficient and effective vaccination campaigns for adapted vaccines in the future.

## Data availability statement

The raw data supporting the conclusions of this article will be made available by the authors, without undue reservation.

## Ethics statement

Ethical review and approval was not required for the study on human participants in accordance with the local legislation and institutional requirements. The patients/participants provided their written informed consent to participate in this study.

## Author contributions

Conceptualization, methodology, formal analysis, investigation, writing the original draft preparation, and visualization: SS and DS. Software and data curation: SS. Validation and writing–reviewing and editing: SS, DS, NL, and LG. Resources, supervision, project administration, and funding acquisition: LG. All authors have read and agreed to the published version of the manuscript.
